# Impact of weather extremes on the spatiotemporal dynamics of visceral leishmaniasis in Brazil

**DOI:** 10.1371/journal.pntd.0013316

**Published:** 2025-07-28

**Authors:** Quinn H. Adams, Emma L. Gause, Rachel E. Baker, Davidson H. Hamer, Guilherme L. Werneck, Lucy R. Hutyra, Kayoko Shioda, Gregory A. Wellenius

**Affiliations:** 1 Center for Climate and Health, Boston University School of Public Health, Boston, Massachusetts, United States of America; 2 Department of Environmental Health, Boston University School of Public Health, Boston, Massachusetts, United States of America; 3 Department of Epidemiology, Brown University, Department of Epidemiology, Providence, Rhode Island, United States of America; 4 Department of Global Health, Boston University School of Public Health, Boston, Massachusetts, United States of America; 5 Center on Emerging Infectious Diseases, Boston University, Boston, Massachusetts, United States of America; 6 Department of Epidemiology, Rio de Janeiro State University, Rio de Janeiro, Brazil; 7 Department of Earth and Environment, Boston University, Boston, Massachusetts, United States of America; Mizan-Tepi University, ETHIOPIA

## Abstract

**Background:**

Vector-borne diseases are highly sensitive to environmental and climatic conditions, which can directly affect vector behavior, parasite development, and transmission dynamics. Identifying the key meteorological drivers of these diseases and understanding the timing of their impacts is crucial for enhancing public health preparedness. This study focuses on visceral leishmaniasis (VL) in Brazil; a parasitic vector-borne disease spread by the bite of infected sandflies whose distribution is heavily influenced by environmental conditions.

**Methodology:**

We analyzed monthly confirmed VL cases from 2007-2022 using distributed lag nonlinear models within a spatiotemporal Bayesian hierarchical model framework to assess the nonlinear, time-lagged associations between locally defined weather anomalies and VL risk across space. We evaluated the exposure-lag-response relationships between anomalies in monthly average temperature, precipitation, and relative humidity; and VL incidence across Brazilian microregions, considering lags ranging from 0 to 4 months.

**Principal findings:**

Among the 53,968 VL cases reported during the study period, the majority occurred in the Northeast and Central North regions. Our model revealed statistically significant nonlinear relationships between meteorological anomalies and VL risk. Associations were most pronounced in rural and deforested microregions, where climatic extremes intensified transmission risk.

**Conclusions and significance:**

This analysis identified an increased VL risk at higher-than-usual temperatures and a lower risk with higher-than-usual humidity and precipitation across various lags. We offer novel foundational insights for the future development of early warning systems, especially relevant to regions like Brazil facing a substantial VL burden.

## Author summary

Visceral leishmaniasis (VL) is a life-threatening disease spread to humans by infected sandflies. VL transmission is influenced by weather patterns, land use, and climate change, but how specific weather events impact disease risk in local areas over short timescales is not well understood. Using spatiotemporal modeling, we captured both the nonlinear and delayed impacts of weather anomalies—such as unusually high temperatures and rainfall—on VL incidence over a range of climatic and environmental conditions. We found that weather anomalies can increase or decrease VL risk depending on timing and location. For example, abnormally high temperatures were associated with higher VL risk 2 months later, while above-average humidity and precipitation contributed to an immediate rise in VL risk, followed by a decrease at longer lags. These associations were more pronounced in rural and deforested regions, where weather anomalies amplified transmission risk. These patterns underscore the necessity for climate-adaptive, spatially targeted public health strategies.

## Introduction

Visceral leishmaniasis (VL) is one of the most deadly parasitic infections globally, posing a significant public health burden, particularly in low-resource settings [1]. VL is a vector-borne disease caused by *Leishmania* parasites, which are transmitted to humans by the bite of an infected female phlebotomine sandfly, primarily *Lutzomiya longipalpis* in Brazil. Of the three forms of leishmaniasis - cutaneous, mucocutaneous, and visceral - VL has the greatest potential to be life-threatening. It is typically characterized by fever, weight loss, splenomegaly, hepatomegaly, and anemia, and, if untreated, is fatal in approximately 95% of cases [2]. VL is a neglected tropical disease, disproportionately affecting vulnerable populations.

Brazil bears the majority of the VL burden in the Americas, accounting for over 90% of cases. This concentrated impact makes Brazil an important focus for research into VL transmission dynamics [2–4]. VL is zoonotic in the Americas and is endemic in many parts of Brazil, with incidence varying both spatially and over time. Although the overall incidence of VL in Brazil has declined over the past two decades, new outbreaks continue to emerge in previously unaffected areas, and the case fatality rate has increased [5,6]. With no available vaccine, early detection and timely treatment are critical for reducing the high mortality associated with VL, especially among vulnerable populations [7]. While VL is treatable, delays in accessing care heighten the risk of treatment failure and death, particularly among individuals with comorbidities. Effective control strategies require proactive planning, yet the lack of robust early warning systems for VL impedes timely responses [7].

Climate change, characterized by rising temperatures, shifts in humidity, altered rainfall patterns, and more frequent extreme weather events, is well-recognized for contributing to the persistence and expansion of vector-borne diseases [8,9], including VL [10], across the globe. Climate variability can promote the spread of disease to new areas by enabling vectors to thrive in environments previously unsuitable for their survival and plays a critical role in shaping the seasonality and intensity of disease outbreaks [8,11–13]. However, the effects of meteorological variability on vector-borne disease risk are complex, often nonlinear, and can be delayed; and the timing of meteorological hazards’ impact on outbreaks is not well understood.

Both the *Leishmania* parasite and sandfly vector are highly sensitive to local weather conditions that are known to influence parasite replication, vector growth rates, and sandfly biology and habitat [2]. For example, air temperature has been found to play a significant role in shaping the geographic distribution of the sandfly vector and has been associated with changes in disease incidence [14–18]. While fluctuations in weather conditions clearly affect VL transmission, the nonlinear and delayed impacts of weather extremes—particularly in the context of shifting climate patterns—remain poorly understood. Exploring these complex relationships is a critical foundational step to developing accurate predictive models and early warning systems. Further, the interplay between changing weather patterns associated with climate change and population-level risk for vector-borne diseases, particularly among neglected tropical diseases in the Global South such as VL, remains underexplored. Comparing current weather conditions to those observed prior to the intensification of climate change could reveal how weather extremes have shifted the distribution of VL.

Rapid, unplanned urbanization and land use changes, such as deforestation, compound the challenges posed by changing weather patterns. These anthropogenic factors can bring humans into closer contact with disease vectors, exacerbating the risk of VL transmission, especially in areas undergoing significant environmental disruption. In Brazil, droughts, farmland scarcity, and famine—especially in the Northeast—have driven large population migrations from rural Brazil to the peripheries of major cities, creating informal and densely populated settlements with many non-immune hosts [19]. Additionally, infrastructure development and large-scale agriculture accelerate deforestation, leading to changes in vector ecology and greater proximity between humans, vectors, and disease reservoirs (commonly domestic dogs in Brazil) in newly deforested areas [20].

This study aims to address gaps in understanding VL distribution by leveraging Bayesian spatiotemporal models to assess how sub-seasonal meteorological anomalies influence VL incidence across Brazil’s diverse climate regions. By examining urbanized and deforested areas, this research explores the distinct ways land use and climate variability interact to shape disease patterns, offering critical insights for developing targeted public health interventions. Brazil’s varied ecology and socioeconomic landscapes offer a unique opportunity to investigate how climate-related stressors influence disease incidence in different settings. This analysis provides critical insights into the complex interplay between climate variability, land use changes, and VL incidence, contributing to a growing body of knowledge that can inform the development of early warning systems for VL. These systems will be instrumental in improving outbreak prediction, guiding public health preparedness, and ultimately mitigating the disease’s impact in both urban and rural settings.

## Methods

### Study area

**Fig 1:**
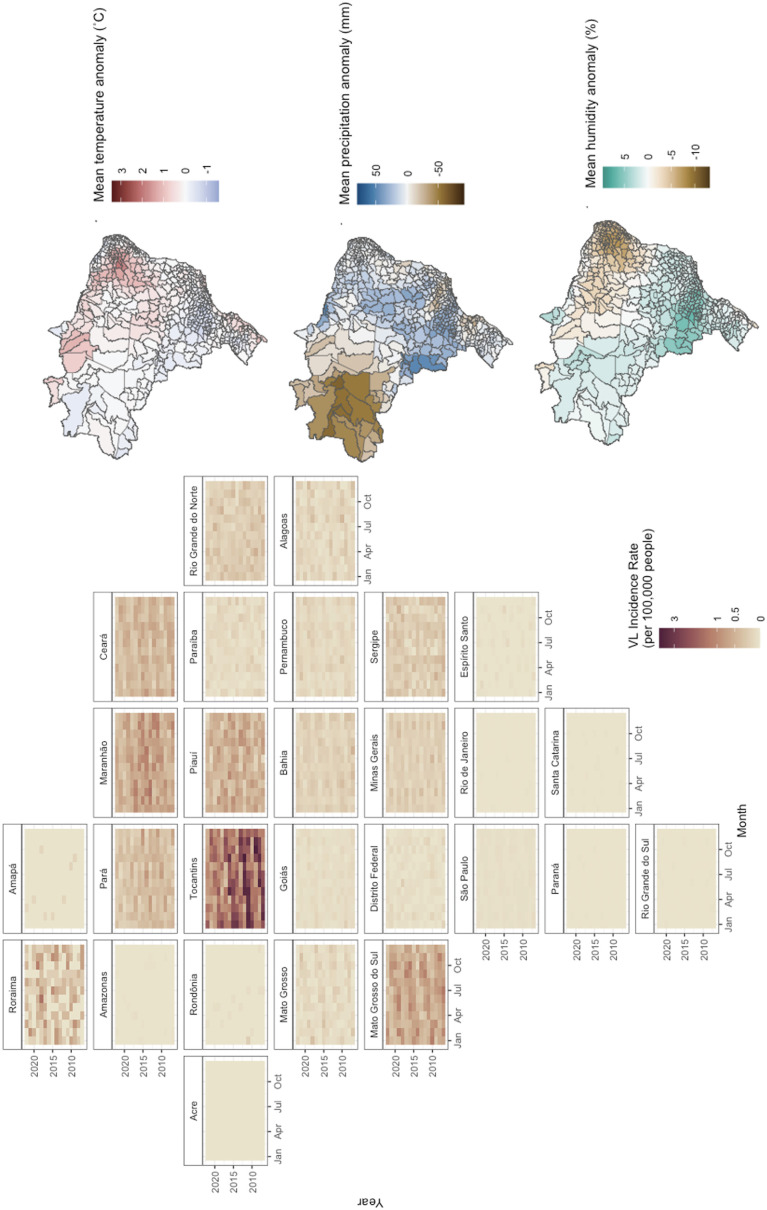
Spatial and temporal distribution of the VL incidence rate per 100,000 people by Brazilian Federative Unit per month and year between 2007 and 2022 (left). Federative units are ordered by geographic location. Average temperature, precipitation, and humidity anomalies by Brazilian microregion comparing the study period (2007-2022) to the long-term reference period (1951-1980) (right). Map shapefiles can be accessed directly from IBGE: https://www.ibge.gov.br/en/geosciences/territorial-organization/territorial-meshes/18890-municipal-mesh.html?edicao=30154&t=acesso-ao-produto.

Brazil is the sixth most populous country in the world with more than 215 million people and spanning 8.5 million square kilometers (3.3 million square miles). Brazil is made up of 27 federative units that are divided into 135 mesoregions, 558 microregions, and 5,570 municipalities nested within nine climate regions. Because 42% of Brazil’s municipalities have fewer than 10,000 inhabitants, we focus this analysis on microregions (Fig 1), which are legally defined geographic areas containing groups of municipalities, to alleviate statistical issues that arise with significant zero-inflated data. A combination of varied elevations, several large climate systems (such as the El Nino Southern Oscillation, the Inter Tropical Convergence Zone, and the South American Monsoon System), and diverse biomes drive Brazil’s distinct climatic and ecologic zones [21]. Average annual temperatures across Brazil have increased by roughly 1˚C (1.8˚F) between 1975 and 2021 [21]. Northern states in Brazil experience substantially higher temperatures and more precipitation on average compared to the southern states [21]. Relative humidity is generally highest in the Northwest and along the eastern coast. Several severe droughts and hydrometeorological events have occurred throughout the Northeast, Amazon, and Southeast regions of Brazil in the past decade, with an increase in the number of meteorological extremes overall [22–24]. There is a projected reduction in annual precipitation over much of Brazil combined with intensified warming patterns under scenarios of continued climate change (RCP4.5 and RCP8.5 radiative forcing scenarios) [25–27].

### Meteorological data

We assessed exposure to several meteorological anomalies to evaluate how deviations from normal weather conditions influence VL dynamics. Gridded monthly mean temperature, humidity, and precipitation data, available at a 9km x 9km spatial resolution, were obtained from ERA5, the fifth generation of the European Centre for Medium-Range Weather Forecasts (ECMWF) reanalysis for climate and weather [28]. For each weather variable, we calculated population-weighted averages by first assigning land-weighted means for each municipality (nested within microregions) where the contribution of a grid cell to the mean value for each municipality is equal to the proportion of the grid cell within the given municipality. We combined these monthly municipality-level mean weather estimates with annual population estimates from the Instituto Brasileiro de Geografia e Estatística (IBGE) to estimate the population-weighted monthly average for each microregion. Population weighting in the context of climate and health is particularly important to accurately represent the weather conditions impacting the population of interest [29]. Monthly long-term averages for each microregion were calculated using 1951–1980 as the baseline period to account for anomalous weather that may be associated with climate change [30]. Monthly climate anomalies for each microregion were calculated using the difference between the monthly measurements between 2007 and 2022 and the long-term monthly averages for temperature, relative humidity, and precipitation.

### VL case data

VL is a nationally notifiable disease in Brazil. We leveraged publicly available data from the Brazilian Ministry of Health Information System for Notifiable Diseases (SINAN), which provides monthly case counts for each of Brazil’s microregions from January 2007 through December 2022 [31]. We aggregated case data by microregion of residence and the month of first symptom. We calculated monthly incidence rates of confirmed VL cases per 100,000 people, using yearly population data in each microregion.

### Statistical analysis

We used a combined modeling approach, leveraging a spatiotemporal Bayesian hierarchical model framework with distributed lag nonlinear models (DLNM) to establish the exposure-lag-response relationship between weather anomalies and the relative risk (RR) of VL incidence, defined below. This method allowed us to capture both the temporal and spatial aspects of VL and its meteorological drivers. Specifically, we explored the relationship between each of the three meteorologic exposures – monthly temperature, precipitation, and relative humidity anomalies – and VL incidence between 2007 and 2022 across 558 Brazilian microregions. We incorporated DLNMs for each meteorologic anomaly in separate models. We explored lags from 0 to 4 months to capture delays in the exposure-response relationship due to VL’s relatively long incubation period, which can be several months long.

We modeled each meteorologic anomaly using a natural cubic spline with knots placed at equal intervals. The RR is defined as the change in the monthly incidence rates of confirmed VL cases per 100,000 people associated with different anomaly exposure levels with 0 as the reference value, indicating no meteorologic change from the baseline. We assumed a negative binomial distribution to account for overdispersion in VL case counts. Spatial and temporal random effects were included to account for unobserved and unmeasured sources of variation. Specifically, spatial random effects with a modified Besag-York-Mollié prior were included to account for unmeasured characteristics that might be similar across adjacent microregions such as weather conditions, sociodemographic characteristics, or VL control interventions [32,33]. These random effects help mitigate potential residual confounding by time-invariant, spatially correlated factors.The parameters and 95% credible intervals were estimated using integrated nested Laplace approximations (INLA) [34,35], a Bayesian inference framework that can effectively capture complex structures in high-dimensional spatiotemporal data [36]. Temporal random effects with a cyclic first-order random walk prior accounted for seasonality and unobserved or unmeasured micro-region level factors that change over time. The models take the following general form:


$$log(Y_{m,t})\;=\;\alpha \;+\;\beta_{m(m)s(t)}+\;\varphi_{m\;a(t)}\;+\;\nu_{m\;a(t)}+\;cb(x_{i\;mt\;},l)$$

Where $log(Y_{m,t})$ represents monthly VL incidence in microregion m at time t. The intercept is represented by α. The state-specific monthly random effects and year-specific spatially unstructured and structured random effects at the microregion level are represented by $\beta_{m(m)s(t)}$, $\varphi_{m\;a(t)}$, and $\nu_{m\;a(t)}$, respectively. The cross-basis function of the DLNM, $\;cb(x_{i\;mt\;},l)$, represents the two-dimensional function describing the weather anomaly (*i*)-VL relationship across the exposure and lag dimensions with lags, *l*, ranging from zero to four months. Lags between zero and four months were selected through sensitivity analyses in which we evaluated lag periods ranging from zero to 12 months. We did not observe significant associations for longer lags. Extending the lag period further complicates the direct link between weather conditions and VL incidence as the biological mechanisms driving disease transmission become less clear over longer timescales. Lags between zero and four months also align with the typical incubation period of several months for symptomatic VL, providing the best balance between statistical association and biological plausibility [1].

Three separate models were used to evaluate the temperature anomaly, relative humidity anomaly, and precipitation anomaly relationships. The reported point estimates represent the median of the posterior distribution and 95% credible intervals represent the 2.5th and 97.5th percentiles of the posterior distribution.

### Urbanization and deforestation

We subsequently explored how the relationship between weather anomalies and VL varied under different levels of urbanization and deforestation.

We evaluated the combined effect of urbanization and monthly temperature anomaly by including a linear interaction between the individual temperature anomaly cross-basis function and a continuous measure of the proportion of residents living in urban areas in each microregion. We acquired urbanization data (proportion of the total population in each microregion living in an urban area) from the IBGE 2010 Population Census. We centered the urbanization variable at the 75th and 25th percentiles to represent highly urbanized and more rural microregions, respectively. The same modeling structure was applied for relative humidity and precipitation anomalies. We also we also included urbanization as an area-level covariate, which allowed the weather-VL association to vary flexibly across levels of urbanization while controlling for structural differences between areas.

We used an analogous approach to evaluate the association between weather anomalies and VL incidence in areas that have and have not experienced deforestation over the study period. Following the methods outlined in Santos et al. (2021) [20], we leveraged land use and land cover data from the Mapbiomas Project and calculated the change in the percent forested area in each microregion between 2007 and 2022. We defined deforested microregions as those with a negative value (i.e.,., lower percent forested area in 2022 compared to 2007). All other microregions were considered to be non-deforested.

While some land use factors, such as urbanization and deforestation, may be associated with long-term climatic conditions, both variables exhibit limited temporal variation over the monthly timescale of this study. Specifically, urbanization is measured annually and the deforestation variable was time-invariant in this study so they are unlikely to confound the within-microregion, over time association between short-term weather anomalies and VL incidence. Instead, these covariates were included to capture variation in baseline VL risk and to allow for the assessment of effect modification.

A flowchart of the methods is available in S1 Fig.

## Results

There were 53,968 confirmed VL cases reported between 2007 and 2022 in Brazil. While the overall incidence has gradually declined, periodic large outbreaks persist (S1 Fig). Seasonal trends were observed across Brazil’s nine climate regions, though with considerable regional heterogeneity (Fig 1). Most VL cases were concentrated in the Northeast and Central North regions (Fig 1), where the climate is predominantly tropical savanna characterized by dry summers and winters. Over the study period, the Northeast and Southern coastlines had the greatest increase in average temperatures relative to the 1951–1980 baseline, while the Amazon experienced the largest reduction in precipitation. In contrast, the Central-West region saw the greatest increase in precipitation, with substantial decreases in relative humidity in the Northeast.

**Fig 2:**
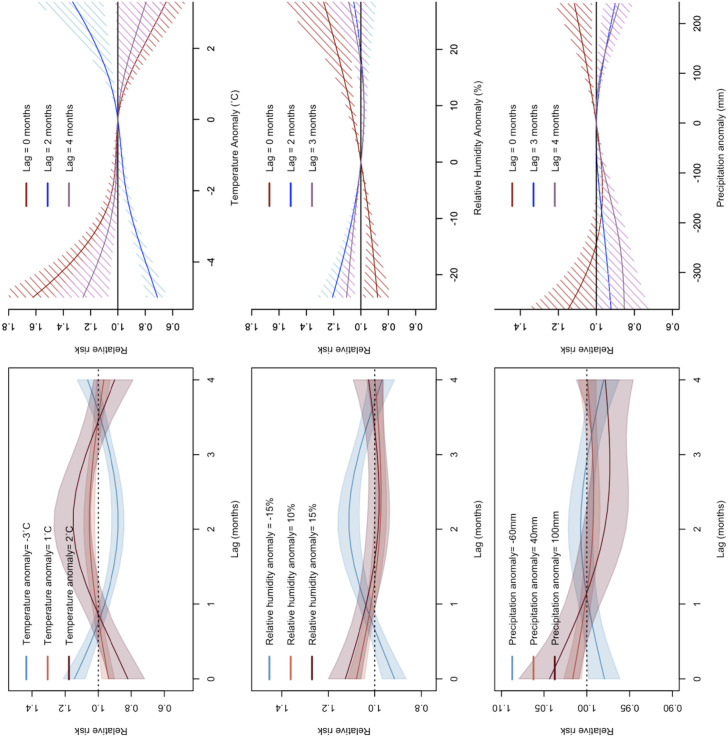
Relationship between weather anomalies and VL incidence in Brazil over different time lags. The left panels show the lag-response functions, and the right panels show exposure-response functions and their 95% credible intervals for temperature (row 1), relative humidity (row 2), and precipitation (row 3) anomalies comparing monthly means between 2007 and 2022 to monthly averages between 1951 and 1980.

Results from the spatiotemporal Bayesian hierarchical model revealed statistically significant nonlinear relationships between meteorological anomalies and VL incidence. Cold anomalies were associated with higher VL risk during the same month, followed by a lower risk 1–3 months later (Fig 2). In contrast, warm anomalies were associated with lower VL risk initially but higher risk 1–3 months after the event. The highest relative risk for VL was observed at a 2-month lag for temperature anomalies. At a 2-month lag, the RR of VL gradually increases with increasing temperature anomalies. The opposite association was observed for lags 0 and 4.

Anomalously high humidity increased VL risk during the same month, while low humidity was linked to a higher risk 1–3 months later (Fig 2). Similarly, higher-than-normal precipitation conditions were associated with an increased VL risk during the same month (Fig 2). Extreme precipitation events, both high (+200 mm) and low (-300 mm), were associated with reduced VL risk after a 4-month lag.

### Weather anomalies

**Fig 3:**
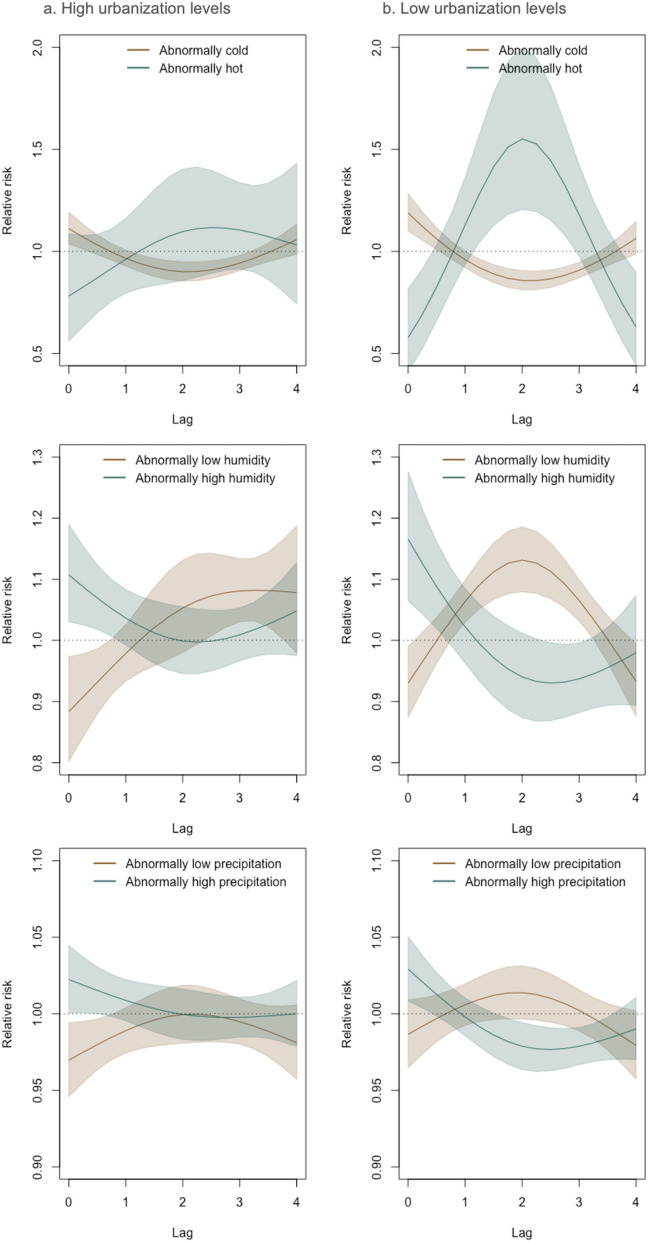
VL lag-response relationship for meteorologic anomalies in urban and rural microregions. Lag-response functions and 95% credible intervals for the relationship between VL incidence and temperature (row 1), relative humidity (row 2), and precipitation (row 3) anomalies at high (upper quartile of residents living in urban areas: 45.7% of microregions) and low (bottom quartile of residents living in urban areas) levels of urbanization. Note: the lags on the x-axes are in months.

### Urbanization

In a further exploration of the association between weather anomalies and VL risk along an urban gradient, we found that weather anomaly-VL associations were stronger in rural microregions, where the relative risk peaked two months after extreme anomalies—such as abnormally high temperatures (+3°C), low humidity (-15%), and low precipitation (-60 mm)—highlighting a potentially heightened vulnerability of rural areas to climatic extremes (Fig 3).

### Deforestation

**Fig 4:**
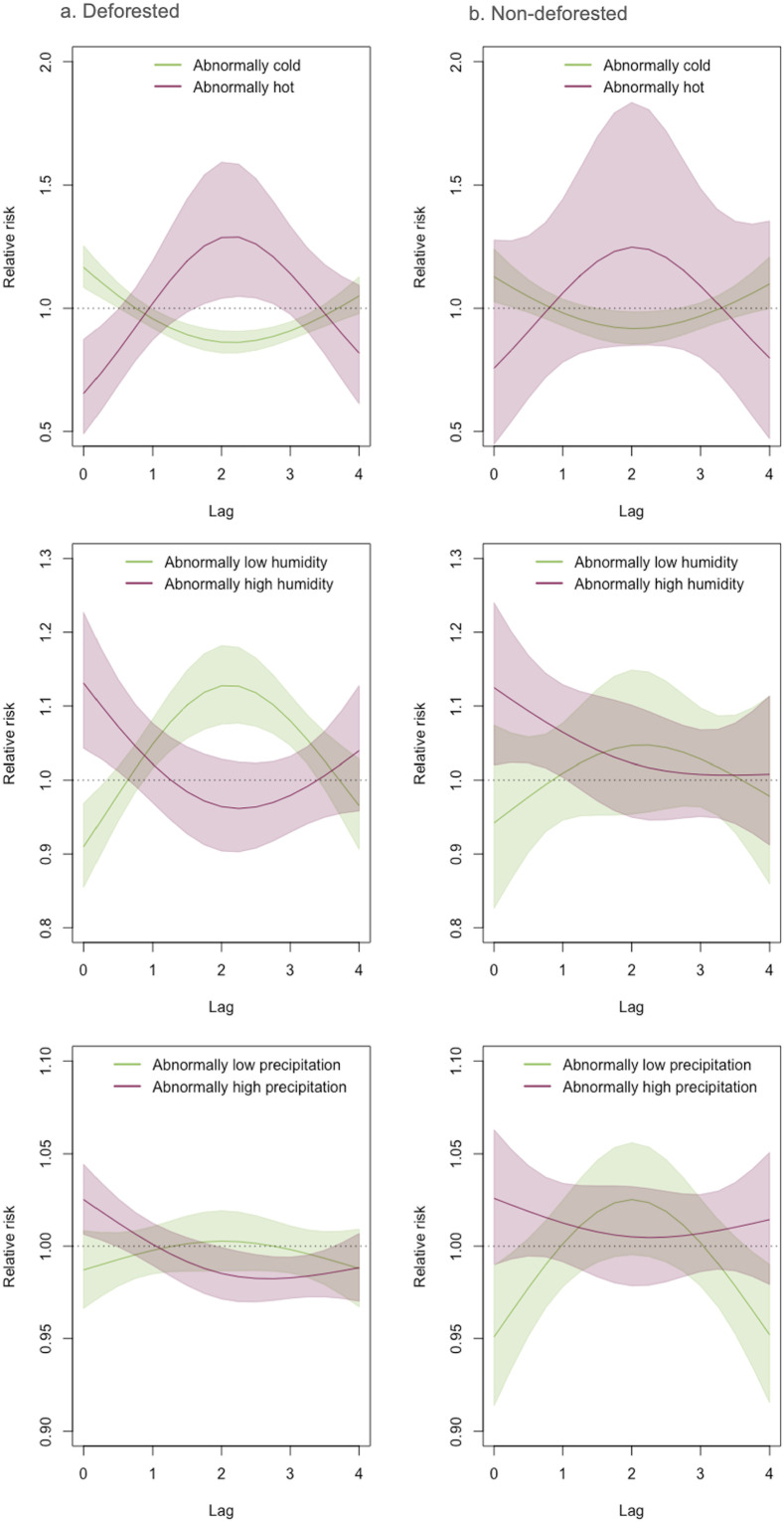
VL lag-response relationship for meteorologic anomalies in deforested and non-deforested microregions. Lag-response functions and 95% credible intervals for the relationship between VL incidence and temperature (row 1), relative humidity (row 2), and precipitation (row 3) anomalies among deforested (43% of microregions) and non-deforested microregions. Note: the lags on the x-axes are in months.

Forty-three percent of microregions experienced deforestation between 2007 and 2022, and in these regions, weather anomalies had an amplified impact on VL risk. In particular, high temperatures (+3°C) and low humidity (-15%) were associated with a marked increase in VL risk two months later, while high precipitation (+60 mm) was linked to a slight reduction in risk at a 2–3-month lag. These effects were most pronounced in deforested microregions, underscoring the role of land-use change in amplifying the impact of climatic variability on VL transmission (Fig 4).

## Discussion

VL transmission is influenced by a complex interplay of environmental, social, behavioral, and climatic factors, which are further complicated by nonlinearities and time-lagged effects driven by both sandfly and parasite development, as well as human incubation periods. In this study, we employed a spatiotemporal modeling approach to unravel the delayed and nonlinear relationships between local weather extremes and VL risk at the microregion level across Brazil. Moreover, we explored how these relationships varied across different levels of urbanization and deforestation. This research provides critical insights into the environmental drivers of VL, which are essential for designing targeted public health interventions.

Our findings indicate that meteorological extremes are associated with both increases and decreases in VL risk at various time lags. For example, we identified an increased VL risk at higher-than-normal temperatures and a lower risk with higher-than-normal humidity and precipitation at various lags, offering novel insights that may be useful for the development of early warning systems. Specifically, abnormally high temperatures relative to monthly averages over the 1951–1980 baseline period resulted in a positive association with VL risk 2 months later. This observation may reflect the facilitation of more rapid vector and parasite development rates and a shorter extrinsic incubation period commonly seen among disease vectors and their pathogens in warmer temperatures [37]. Unexpectedly, we observed a slightly higher risk of VL at anomalously cool temperatures at lags 0 and 4 months. This is consistent with entomological studies showing that the *L. infantum* parasite can successfully establish in the sandfly midgut at both low and high temperatures [38]. However, sandfly development tends to be slower under colder conditions, which may not have been fully captured in our 4-month lag period. Further exploration of these dynamics, possibly through extended lag periods or more theoretical modeling approaches like compartmental models, could provide deeper insights into these temperature-related mechanisms.

Anomalously high humidity levels were positively associated with VL risk during the initial 0–1 months. During this period, VL risk gradually increased with increasing relative humidity anomalies. In contrast, longer lag times were associated with an increased risk of VL at abnormally low humidity levels, suggesting an intricate balance where moisture conditions alter sandfly activity and influence human behavior at various levels and over different time courses. High levels of precipitation were associated with an immediate increase in VL risk during the same month (lag 0) and a slightly lower VL risk roughly 3 months later. Previous studies have proposed that rainfall can enhance sandfly density by increasing the availability of larval resting sites rich in organic material, whereas excessive rainfall may reduce vector density by destroying breeding sites [39]. However, associations with precipitation are likely to be context-specific, depending on regional ecological dynamics.

The associations between weather extremes and VL risk were especially pronounced in more rural and deforested microregions. Several mechanisms may explain the more significant climate impacts in these areas. For example, land-use changes, such as deforestation, may amplify the effects of climatic factors on VL transmission by forcing vectors and animal reservoirs into closer proximity with human populations, thereby enhancing transmission potential. In contrast, we observed a less pronounced relationship between meteorological extremes and VL risk in urban areas. There are several possible reasons why the relationship between weather extremes and VL risk may be stronger in rural areas. For example, the *L. longipalpis* species is known to rapidly adapt to urban environments, where they may find alternative microenvironments that favor vector proliferation, such as in the accumulation of organic waste [40]. Therefore, urban VL transmission may be less dependent on weather-related factors and more influenced by socio-environmental characteristics such as sanitation, poor housing conditions in unplanned urban areas, or larger proportions of the population with chronic conditions that heighten susceptibility to VL. These results point to the critical and complex role that anthropogenic environmental modifications play in shaping the epidemiology of VL.

These findings should be interpreted in light of several limitations. First, the spatiotemporal resolution of our analysis—at the monthly and microregional levels—does not fully capture localized or rapid changes in weather conditions that could affect VL transmission. Second, the reliance on reported VL cases via passive surveillance may introduce biases due to underreporting or inconsistencies in diagnostic capacity, particularly in rural or resource-limited areas, potentially affecting the accuracy of our risk estimates. Similarly, a large proportion of VL infections remain asymptomatic for the duration of infection. Specifically, the estimated ratio of asymptomatic infection to clinical manifestation in Brazil has been demonstrated as 18:1 [41,42.] The risk of progression to symptomatic VL or relapse even after successful treatment is substantially higher in individuals with HIV or other conditions associated with immunodeficiency and can occur years to decades after the initial infection [43]. The rate of progression to symptomatic VL among these populations is expected to be relatively low, but may also overestimate the association between weather and VL at the chosen time lags. Third, without access to sandfly density data at this scale across Brazil, we were unable to elucidate the impacts of meteorological factors on the sandfly vector as a critical mediating factor. Finally, there are several mechanisms by which temperature and humidity may interact to exacerbate or attenuate the survival, reproduction, and transmission capacity of disease vectors and, consequently, VL transmission [44]. However, our analysis focused on weather anomalies as opposed to absolute weather conditions and notable associations for these interactions are unexpected when examined at the microregion and monthly levels. Similarly, inference for interacting exposures at different time lags is particularly complex given VL’s long incubation period, which is typically between two and six months [1].

Nonetheless, this study expands the limited knowledge of the associations between various meteorological characteristics and VL in Brazil from a spatiotemporal perspective. This modeling framework has been successfully implemented among other climate-sensitive infectious diseases and can therefore have implications for research on other disease-environment systems [45–47]. By integrating diverse data sources and advanced spatiotemporal analytical methods, our study provides a comprehensive understanding of how meteorological factors influence VL risk across a range of socio-environmental contexts. A thorough understanding of the spatiotemporal dynamics of VL associated with weather extremes and their interactions with urbanization and deforestation enhances the predictive capacity for the timing and intensity of VL outbreaks.

Results from this study underscore the importance of developing proactive public health strategies that account for both climatic variability and land use changes. Effective VL control requires integrated strategies that target both vectors and address broader policy measures to manage urban growth and deforestation in ways that mitigate VL risk. Ultimately, integrating meteorological insights into public health planning offers a path toward more resilient health systems capable of addressing the challenges posed by vector-borne diseases under changing environmental conditions. The relationship between meteorological covariates and VL incidence may form a strong basis for VL early warning systems to improve planning and control for VL outbreaks. These early warning systems could guide vector control efforts, optimize resource allocation, and ultimately mitigate VL incidence, especially in vulnerable regions undergoing rapid environmental change.

## Conclusion

This study demonstrates that VL incidence in Brazil is influenced by climatic extremes measured by temperature, humidity, and precipitation anomalies, with varying effects based on time lags and regional contexts of urbanization and deforestation. These findings underscore the critical role of meteorological information in enhancing public health planning and enabling more targeted, proactive interventions to mitigate VL risk. Moreover, addressing environmental changes such as deforestation is vital for reducing disease transmission and strengthening resilience to vector-borne diseases in Brazil and other endemic regions. Our results provide a strong foundation for developing integrated public health strategies that account for climate variability, land-use changes, and disease management, offering a pathway to more effective control of VL outbreaks.

## Supporting information

S1 Fig Flowchart illustrating the methodological approach for analyzing the relationship between meteorological data, VL case data, and environmental factors using Bayesian Hierarchical Models with Distributed Lag Nonlinear Models (DLNM). The flowchart includes data collection, processing, and analytical steps leading to the generation of results and insights.
(TIF)
