# Supplementary figures and images for: Impact of weather extremes on the spatiotemporal dynamics of visceral leishmaniasis in Brazil
Source: PLoS Negl Trop Dis. 2025 Jul 28;19(7):e0013316. doi: 10.1371/journal.pntd.0013316 (PMC12303289; doi:10.1371/journal.pntd.0013316)

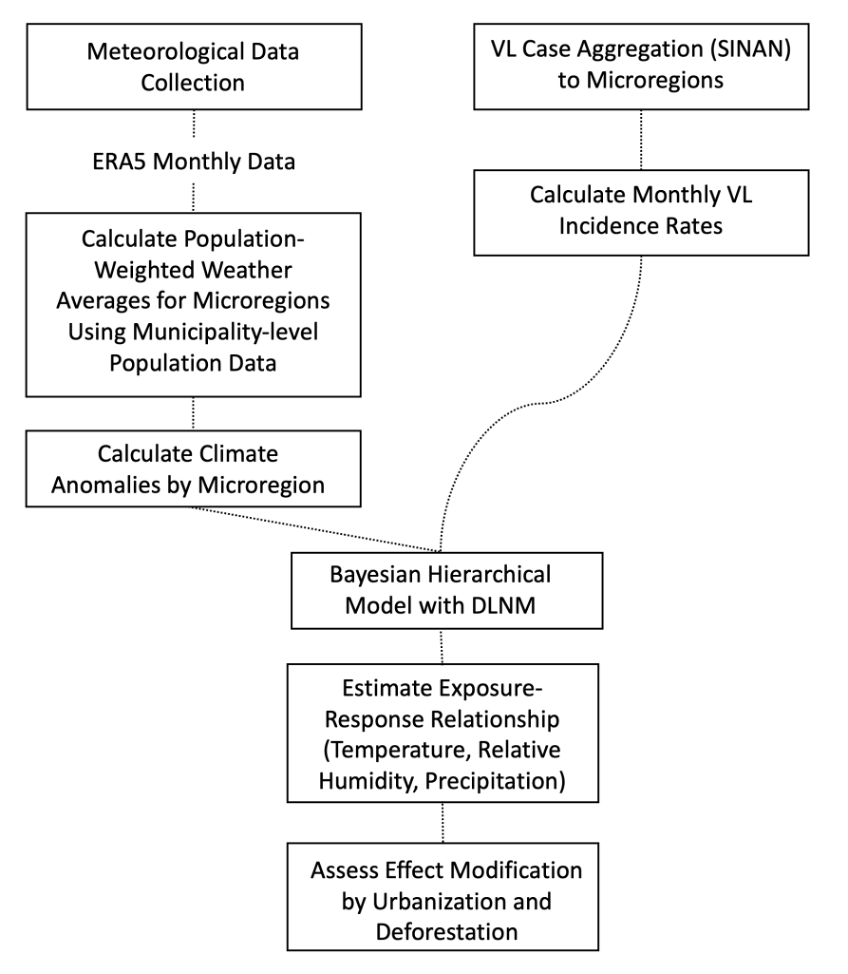

Supplement: S1 Fig — The flowchart includes data collection, processing, and analytical steps leading to the generation of results and insights. (TIF) [file pntd.0013316.s001.tif]
